# Activated carbon as catalyst support: precursors, preparation, modification and characterization

**DOI:** 10.3762/bjoc.16.104

**Published:** 2020-06-02

**Authors:** Melanie Iwanow, Tobias Gärtner, Volker Sieber, Burkhard König

**Affiliations:** 1 Fraunhofer Institute for Interfacial Engineering and Biotechnology IGB, Bio-, Electro- and Chemocatalysis BioCat, Straubing Branch, Schulgasse 11a, 94315 Straubing, Germany; 2 Department of Chemistry and Pharmacy, University of Regensburg, Universitätsstraße 31, 93040 Regensburg, Germany; 3 Technical University of Munich, Campus Straubing for Biotechnology and Sustainability, Schulgasse 16, 94315 Straubing, Germany

**Keywords:** activated carbon, catalysis, characterization techniques, metal supported on carbon catalysts, preparation methods

## Abstract

The preparation of activated carbon materials is discussed along selected examples of precursor materials, of available production and modification methods and possible characterization techniques. We evaluate the preparation methods for activated carbon materials with respect to its use as catalyst support and identify important parameters for metal loading. The considered carbon sources include coal, wood, agricultural wastes or biomass as well as ionic liquids, deep eutectic solvents or precursor solutions. The preparation of the activated carbon usually involves pre-treatment steps followed by physical or chemical activation and application dependent modification. In addition, highly porous materials can also be produced by salt templating or ultrasonic spray pyrolysis as well as by microwave irradiation. The resulting activated carbon materials are characterized by a variety of techniques such as SEM, FTIR, nitrogen adsorption, Boehm titrations, adsorption of phenol, methylene blue and iodine, TPD, CHNS/O elemental analysis, EDX, XPS, XRD and TGA.

## Introduction

Support materials for metal catalysts allow the dispersion and stabilization of small metal particles on a surface. Compared to the bulk metal, these catalyst preparations present a higher surface area of catalytically active atoms [1]. The use of activated carbon as support for metal catalysts shows several advantages compared to other support materials. The carbon surface is inert, especially in strongly acidic and basic conditions, the pore size distribution and the chemical properties on the surface can be adjusted (polarity and hydrophobicity) according to the envisaged application. In addition, metal particles can be recovered simply by burning the carbon support [1–2]. Thus, porous carbon materials represent a large part of the supporting materials for the preparation of heterogeneous catalysts. Nevertheless, only a small amount of the worldwide produced activated carbon (<1%) is used as catalyst support. A possible reason may be the lack of reproducibility due to inconsistent carbon precursor compositions [2].

In general, activated carbon is an amorphous carbon modification with a high surface area and a well-developed porosity, which can be produced from a variety of carbon sources [3]. The preparation of activated carbons is already broadly covered in the literature. Therefore, we limit our brief overview to selected examples. Emphasis is given to carbon precursors, preparation and modification methods, characterization techniques and metal loading on the carbon materials.

## Review

### Precursor materials for activated carbon production

Many cheap raw materials with high carbon content can be used for the production of activated carbon [4]. Fossil and renewable sources for the preparation of activated carbon are discussed in this part of the review, as well as special precursor solutions or ionic liquids and deep eutectic solvents as non-conventional precursor materials. The properties of the resulting activated carbon depend, in addition to the type of precursor material, on the preparation or activation method and the modification used.

#### Coal

In the beginning of the 1990s, 360 kilotons activated carbon were produced, whereby 42% were based on coal as precursor due to the availability and low coast of coals such as brown coals, bituminous coals, petroleum cokes or anthracites. The coals should have a low mineral matter content and thus, a low ash content [4–5]. Bituminous coal-based activated carbons result in a well-developed porous structure due to the presence of primary pores in the coals. However, the size of these pores is very small [5]. Activated carbons prepared from bituminous coals are more durable compared to other coal-based carbons [6]. Petroleum coke as byproduct of the refinery industry, show a high carbon content, low amount of ash and is widely available [7]. Anthracites are very suitable precursors for activated carbon (AC) preparation, since they are high-rank coals (high C to H atomic ratio without carbonization) and show a non-negligible volume of very fine micropores [8–9].

Various working groups investigated the preparation of activated carbons by different methods from coal precursors. Yang et al. synthesized nitrogen-doped activated carbon from petroleum coke for an enhanced CO_2_ capture [7]. Pietrzak et al. used high volatile bituminous coals, brown coals and anthracites for modified activated carbon preparation [5,10–12]. Lillo-Ródenas et al. investigated the chemical activation with sodium or potassium hydroxide by the use of an anthracite precursor [8,13–14].

#### Wood

Non-fossil precursors for the preparation of activated carbons are of great interest due to an increasing demand of these materials. Wood and the lignocellulosic wastes from forestry and agriculture are well-suited for this purpose [15]. Wood is mainly composed of cellulose (40 to 55 wt %), hemicelluloses (mostly xylan in hardwoods with 20 to 35 wt %) and lignin (18 to 35 wt %). Cellulose maintains the structure of the cell walls of plants and is the most abundant raw material with a production of 10^11^–10^12^ tons per year, followed by lignin as second most abundant raw material [16]. Lignin is a three-dimensional phenolic polymer and is responsible for the cementation of cellulose fibres in plants [17]. Hemicelluloses, predominantly xylan, are non-cellulosic polysaccharides with a comparable low molecular weight [15]. Khezami et al. investigated the preparation of activated carbon from wood and its main components: cellulose, xylan and lignin [15]. Suhas et al. reviewed the use of cellulose as well as lignin for activated carbon preparation in detail [16–17]. Hameed and co-workers prepared high surface area AC from wood sawdust [18].

### Agricultural waste/ biomass

The use of agricultural waste as precursor for activated carbon materials is summarized in a variety of reviews [3,19–24]. Any low cost lignocellulosic materials with high carbon content are of great interest as starting material [22]. An exemplary overview of agricultural byproducts and waste used for the production of activated carbon materials is shown in Table 1.

**Table 1:** Exemplary overview of agricultural waste sources for activated carbon production.

Carbon source	References

straw	[25–29]
rice husk	[23,30–33]
bagasse	[30,34–37]
miscanthus	[38–39]
bamboo	[40–42]
cotton residues	[43–44]
nut shells	[45–49]
fruit pits	[49–53]
fruit seeds	[54–56]
fruit peels	[57–59]
coconut shells	[60–63]
olive stones	[64–66]
sunflower seed oil residues	[67–68]
coffee residue	[69–70]
corn cobs	[71–72]
oil palm residues	[73–75]
rotten strawberries	[76]

According to Ioannido et al., the composition and structure of the used raw material determines the reactivity during pyrolysis and activation steps and thus, the resulting elemental composition. They concluded that pyrolysis of agricultural waste provides up to twice the amount of char obtained from wood. Different starting materials yield activated carbons with different ash contents or BET surface areas. Nutshells and cherry stones show for example less ash content compared to grape seeds. Olive wastes and bagasse results in activated carbons with high surface areas, whereas straw is less suitable to produce high surface areas [19]. Yahya et al. mentioned that the yields of the activated carbons prepared from these residues is lower compared to anthracite or coal as starting materials. Nevertheless, high volatile matter content in the biomass are advantageous for the production of porous activated carbon materials as well as the low cost of the agricultural waste [22].

#### Ionic liquids and deep eutectic solvents

Zhang et al. demonstrated the preparation of carbon materials with high surface areas from protic ionic liquids and salts. The precursors have low-molecular weights, are available and cheap. Preparation of the carbon materials is simple: Neutralization of the nitrogen-containing bases, e.g., phenanthroline or 3-cyanopyridine with sulfuric acid and subsequent removal of the solvent leads to the desired protic ionic liquids and salts. Carbonization at 1000 °C results in the final carbon materials with high nitrogen content without further modification. The basic components of the protic ionic liquids influence the yield of the materials. Thermally stable benzene moieties increase significantly the amount of carbon material produced, whereas mixtures based on amines or heterocycles decrease the yields [77]. The salt templating method developed of Antonietti and co-workers produces carbon materials with high surface areas from ionic liquids. A defined salt mixture was added to the ionic liquids 1-butyl-3-methylpyridinium dicyanamide (Bmp-dca, N-doped materials) or 1-ethyl-3-methylimidazolium tetracyanoborate (Emim-tcb, N- and B-doped materials) and the mixture was heated under nitrogen atmosphere. Removal of the salt by immersing in water for several hours, filtration and drying in vacuum leads to the final carbon material [78]. Iwanow et al. investigated deep eutectic solvents as raw materials for activated carbon production. They dissolved the metal salts already in the low melting mixtures before pyrolysis and prepared carbon-supported metal catalysts in one-step. Nevertheless, the surface area of these materials is much lower compared to the conventional activated carbons, but high nitrogen contents are obtained depending on the composition of the deep eutectic solvents [79].

#### Precursor solutions

Xu et al. used energy-rich carbon precursors for the spherical carbon preparation via ultrasonic spray pyrolysis. Lithium, sodium or potassium propiolates are one class of such energy rich materials and exhibit leaving groups such as CO, CO_2_ or C_2_H_2_ eliminated by decarbonylation or decarboxylation. Poly(propiolate) salts are formed by polymerization of the starting materials after heating. The different cations of the propiolates cause changes in the thermal behavior of the starting materials since different temperatures are required for the decomposition or different amounts of gases are released. Alkali salts of acetylene dicarboxylic acid can also be used as precursor for the preparation of carbon spheres. The structure and morphology of the carbon spheres can be influenced by the used alkali salts [80].

### Activated carbon preparation

The following chapter summarizes the different preparation methods for activated carbon materials. Depending on the carbon source, different pre-treatment steps are required before the carbonization/pyrolysis and activation of the precursor materials can be performed. In addition to the most widely used method of physical or chemical activation, specialized methods such as salt templating and ultrasonic spray pyrolysis are presented.

#### Pre-treatment

Different pre-treatment steps are necessary before carbonization or activation of the carbonaceous precursors. The use of biomass as carbon precursor requires often additional washing steps to remove impurities [30]. Drying at ≈100 °C for a defined time removes the free moisture in the material, which could affect the carbonization step [81]. A defined and standardized starting material size of the raw materials is also essential for the activated carbon production process and is obtained by milling and sieving of the carbon precursors [30,45,81–82].

#### De-ashing/demineralization

Activated carbons contain different ash contents due to mineral components in the raw materials, which affect the chemical properties of the prepared materials. For catalytic applications, only activated carbons with the lowest possible ash contents can be used. Prior to the production of activated carbons from the precursor materials, the amount of ash and minerals in the materials is reduced by leaching with acidic or basic solutions [83]. Samples are mostly demineralized by concentrated hydrochloric and hydrofluoric acids according to the Radmacher and Mohrhauer method [10]. Dofour et al. described the procedure in detail. The stepwise treatment with hydrochloric acid, hydrofluoric acid and again hydrochlorid acid removes the metal oxides and silica in the samples and is a comparatively soft method for the carbon material. The treatment with HNO_3_ is another possibility for the removal of the mineral contents; however, this method causes the oxidation of the material and thus, produces new oxygen-containing surface functionalities [84].

#### Physical activation

Physical activation of carbon materials is a two-step process. After preparation of char by carbonization of the precursor materials for a certain time at a defined temperature under inert gas atmosphere, steam, air or CO_2_ activate the materials at higher temperatures (800–1000 °C) to form a porous structure according to the Equations 1–3 [81,85–86].

[1] $${\text{C + O}}_{\text{2}}\to {\text{CO}}_{\text{2}}$$

[2] $${\text{C + CO}}_{\text{2}}\to 2\text{ CO}$$

[3] $${\text{C + 2 H}}_{\text{2}}\text{O}\to {\text{CO}}_{\text{2}}+{\text{2 H}}_{\text{2}}$$

It is necessary to eliminate a high amount of internal carbon for the formation of a well-developed and highly porous carbon structure [6]. In general, the physical activation of carbon materials has the advantage over chemical activation to avoid impurities or additives in the final materials from the incorporation of the activating substances [16].

Several literature reports show a higher reactivity of steam as mild oxidizing reagent compared to carbon dioxide. Nevertheless, no clear tendencies were found regarding the pore development [87–91]. Rodríguez-Reinoso et al. and Zamora and co-workers investigated the influence of the different physical activation gases on the development of porosity from olive stone-based chars. They concluded that activation with steam results in activated carbon materials with lower micropore volumes and wider pore size distributions (higher amounts of meso- and macropores are formed) compared to carbon dioxide [64,87]. Kalderis et al. prepared activated carbons from bagasse and rice husk by physical and chemical activation. They found that physical activation results in significantly lower surface areas compared to the surface obtained by chemical activation with zinc chloride at the same temperature [30].

#### Chemical activation

Chemical activation is a one-step method. Impregnation or mixing of the carbon precursor with the activating agent and subsequent carbonization of that mixture leads to highly porous activated carbon materials [8,92]. The activation agents promote a cross-link formation due to their dehydration properties, which causes a rigid matrix. This structure is less susceptible to volatile loss and volume contraction during the carbonization resulting in higher activated carbon yields since no carbon burn-off is necessary [6,85]. Additional advantages of the chemical activation are lower temperatures for pyrolysis, the formation of very high surface areas and it is possible to control the development of microporosity, e.g., a narrow pore size distibution can be obtained [8,15]. Various parameters influence the formation of porosity during the chemical activation process. A few examples for the different preparation conditions are the choice of the activing agent (KOH, NaOH, ZnCl_2_, H_3_PO_4_, MgCl_2_, AlCl_3_, K_2_CO_3_, etc.), the impregnation technique used or physically mixing processes, the activation reagent to carbon precursor ratio, the flow of inert gas during carbonization and the pyrolysis temperature and time [13–14].

Highly developed pore structures are obtainded by chemical activation of carbon precursors with hydroxides [9,14]. Song et al. investigated the activated carbon preparation by chemical activation with KOH from corn cob. They found that KOH plays a crucial role in the formation of porosity. Metallic potassium is formed during the carbonization of the carbon precursor, which intercalates in the carbon structure and is responsible for further release of carbon dioxide, carbon monoxide and hydrogen [71]. According to Marsh and Rodriguez-Reinoso, the activating agent reacts with the formed char and not directly with the carbon precursor to form a porous structure [56,93]. Hsu et al. studied the preparation of highly porous activated carbons from bituminous coal by chemical activation with ZnCl_2_, H_3_PO_4_ and KOH. The choice of the activating reagent influences strongly the activated carbon properties. Higher yields are obtained by activation with ZnCl_2_ and H_3_PO_4_ compared to KOH, whereas less porosity is developed. The acidic character of the activation reagents ZnCl_2_ and H_3_PO_4_ seems to be suitable for the development of large pore structures in the carbon source [6]. Diamadopoulos and co-workers observed also the production of smaller surface areas by H_3_PO_4_ activation of bagasse and rice husk due to a reaction of the activating reagent with the carbon precursor. Thereby, phosphate esters or polymerization byproducts are formed, which are strongly bonded to the carbon matrix and are not removed by the subsequent washing step [30].

#### Salt templating

Fechler et al. developed the salt templating method for the preparation of highly porous functional carbon materials using ionic liquids as carbon source. Inorganic non-carbonizable salts were mixed with the carbon precursor and elevated temperatures lead to condensation and scaffolding of the carbon source by the presence of the molten salt. The aim of the method is to retain as long as possible the miscibility of the carbon precursor and salt melt during the reaction. After a washing step to remove the salt from the carbon materials, high specific surface areas are obtained with pore sizes corresponding to the salt clusters and salt percolation structures. The polarizability can be adjusted by selection of the cation size and counterion, which influences the pore size and the miscibility during the production process [78,94].

#### Ultrasonic spray pyrolysis (USP)

**Figure 1:**
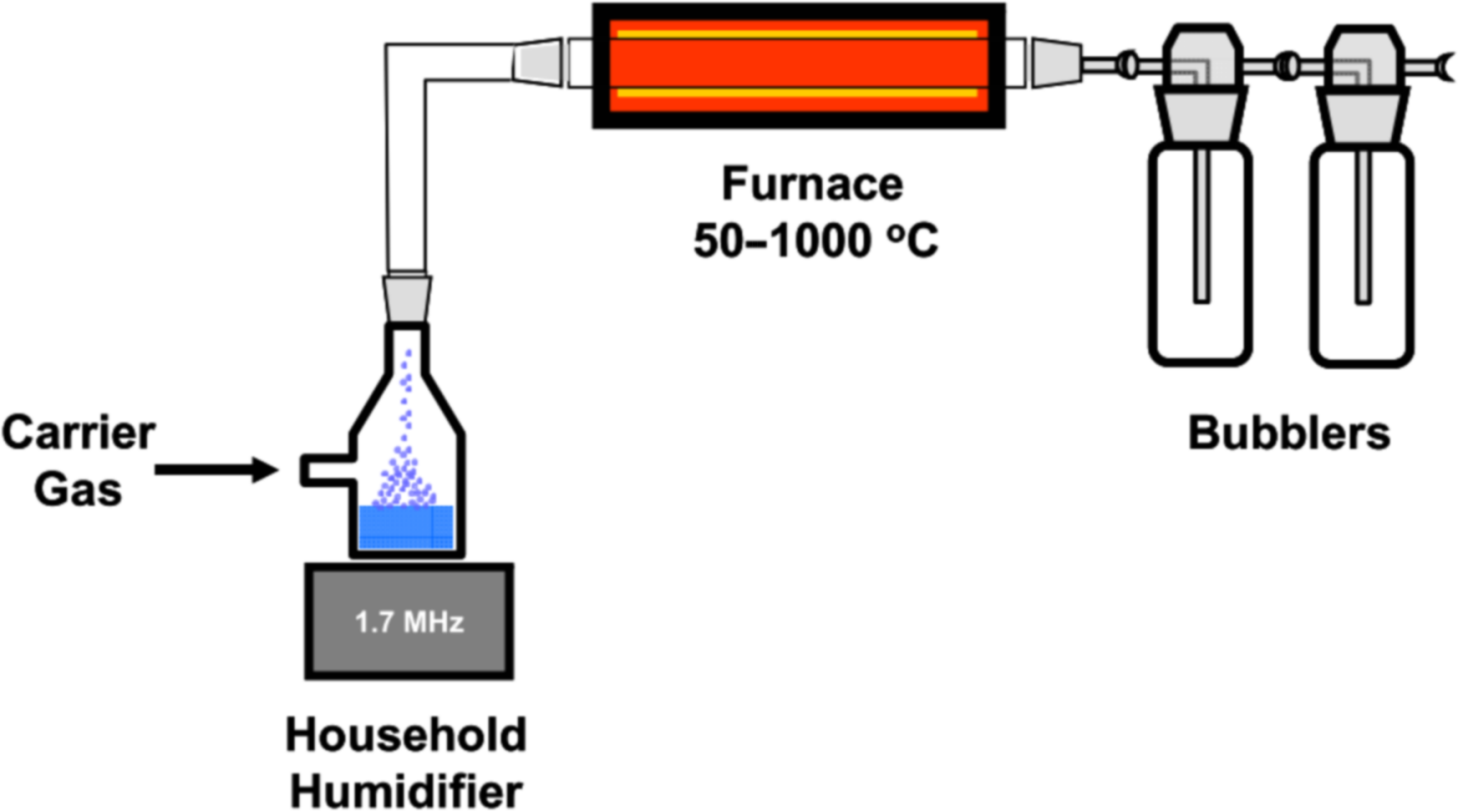
Experimental setup of ultrasonic spray pyrolysis. Reprinted with permission from [95], copyright 2006 The American Chemical Society.

The USP method for continuous preparation of meso- and macroporous carbon spheres was used by Skrabalak et al. Ultrasonically nebulization of a precursor solution (carbon source and inorganic salts) by a humidifier results in a mist of micron-sized droplets. These droplets are transported into a furnace by an inert gas stream, where the solvent evaporates and the precursor decomposes. The formed carbon sphere/salt composites are collected in water bubblers. The salt is dissolved in the collection solvent and byproducts either remain in the solvent or were removed by the gas stream resulting in the desired porous carbon spheres (Figure 1) [95].

Suslick and co-workers used the USP process for the preparation of well-dispersed iron impregnated porous carbon microspheres. An iron source (FeCl_3_ or Fe(NO_3_)_3_ is already added to the precursor solution consisting of sucrose as carbon source and NaCl or NaNO_3_ as inorganic salt. The pyrolysis of the precursor solution leads to dehydration of carbon as well as iron salt conversion to crystalline or non-crystalline iron species depending on the production conditions. The porosity of the carbon spheres is induced by either aromatization of carbon around an in situ template, in situ chemical activation or gasification of carbon [96]. Xu et al. found that the morphology of the porous carbon materials prepared from propiolate salts with USP are dependent on the choice of starting materials. The thermal decomposition behavior of the precursors, and thus the resulting morphology of the carbon materials is influenced by the propiolate cations [80].

#### Spherical carbons

The preparation of spherical mesoporous carbon particles as catalyst support with high surface areas, controllable particle sizes and large uniform pores received much attention [97–98]. One possibility to prepare such carbon spheres with defined particle sizes and pore structures is the nanocasting strategy using silica scaffolds as shown by Fuertes [99]. Nevertheless, due to the complex and high-cost preparation of these carbon materials (preparation of the solid scaffolds, pyrolysis of the carbon precursors in these templates and finally, the selective removal of the silica template) and the risk of structure and morphology defects by the harsh carbonization and template removal processes, this method is industrially infeasible [97].

Carbon spheres are also prepared by hydrothermal treatment of aqueous low cost biomass, such as lignocellulosic materials, or carbohydrate precursor solutions at defined temperatures in closed systems [100–101]. The proposed mechanism of the formation of carbon spheres seems conform to the LaMer model and starts with a polymerization step of the carbohydrate monomers and followed by a carbonization step when the nucleation is caused by the supersaturation of the solution. The resulting nuclei grow uniformly until the final size is obtained depending on the growth parameters [102]. Linares-Solano and co-workers activated the resulting carbon spheres to develop different textural properties by maintaining the morphology. Surface areas higher than 3100 m^2^ g^−1^ could be synthesized [98].

Yan et al. presented another method for the preparation of spherical carbon materials by aerosol-assisted self-assembly using amphiphilic triblock copolymers as template and low-molecular weight soluble phenol resin as carbon source. The amphiphilic surfactant influences the pore size and mesostructure of the resulting spherical carbons. Finally, the template is removed by calcination [97].

#### Comparison of conventional and microwave heating

Conventional heating for physical or chemical activation of the precursor materials has several drawbacks such as the non-uniform heating of the samples or a high energy demand due to long carbonization and activation times at high temperatures [103–104]. Microwave irradiation is a promising alternative with some advantages. In contrast to conventional heating, which is based on the convection mechanism involving conduction and radiation, the sample can be heated uniformly and contactless by the heat generated from electromagnetic energy, resulting in significant time reduction and therefore energy savings [103]. The major problem of microwave heating is that the carbon sources are poor receptors for the irradiation, thus activation agents are necessary as heat carriers and for promotion of porosity [104].

Wang et al. prepared activated carbons with high surface areas by microwave-induced ZnCl_2_ activation within minutes. The porosity of the materials can be tailored by the carbon precursor to ZnCl_2_ ratio and the microwave irradiation time [104]. Foo et al. investigated the activated carbon preparation by microwave heating with K_2_CO_3_ activation from wood sawdust. They obtained highly porous activated carbons by varying the impregnation ratio, microwave power and irradiation time. They concluded that the high surface areas are formed due to opening of previously inaccessibly pores and the additional creation of new pores by the interior and volumetric heating of microwave radiation [18]. Lin and co-workers compared the activated carbon preparation with KOH activation by conventional and microwave heating. The microwave-induced materials showed higher surface areas compared to those prepared by conventional heating using the same precursor to activating agent ratio [105].

### Activated carbon modification

Depending on the starting materials or preparation methods used, various modification treatments can be performed to functionalize the surface of the activated carbons according to the subsequent use of the materials.

#### Surface area and porosity

In general, a high surface area and well-developed porosity of activated carbons are beneficial for the use as catalyst supports to obtain a highly dispersed loading of metal particles on the surface. The size of the pores is also important. Highly porous activated carbons with narrow micropores can block active centers being not available for the reactants [2]. The surface area and development of porosity (amount of porosity, pore size and shape) of the activated carbons can be influenced by the preparation conditions.

Lyubchik et al. used different chemical (HClO_4_ or Mg(ClO_4_)_2_) and physical (CO_2_) activation methods for the modification of the porosity of anthracite-based activated carbons. The final pore size distribution (mainly microporous or mainly mesoporous) depends on the choice of the activation agent, the treatment time and temperature and the initial textural properties of anthracite as carbon source [106]. Wang et al. found that acidic treatments generally enhance the surface area and porosity of activated carbons, since the inorganic impurities in the materials were removed. Hydrofluoric acid modification showed the greatest enhancement of the surface area and porosity [107]. However, the surface area and developed porosity of the activated carbon materials is only one parameter affecting the material application as catalyst support. Another important characteristic is the chemical composition of the activated carbon surface [2].

#### Chemical surface properties

The chemical properties of the carbon surface influence the acid–base and hydrophilic character and thus can affect the preparation of carbon-supported catalysts. Different types of active phase–support interactions can be induced by the introduction of heteroatoms on the carbon surface, which is only marginally possible in other catalyst supports, e.g., silica or alumina [2]. Depending on the application of the carbon-supported catalysts, different possibilies are available for the modification of the properties on the surface of the activated carbon materials.

**Oxygen-containing surface groups:** The amount and composition of oxygen-containing surface groups can be influenced by treatment with different oxidants such as H_2_O_2_, HNO_3_, oxygen/ air, ozone or NaOCl. Thereby, the acidic or basic behavior and the resulting surface chemistry of the activated carbons are determined.

Jaramillo et al. investigated the influence of different oxidizing reagents on the activated carbon materials prepared from cherry stones. Different amounts of oxygen functional groups were found on the surface of the materials depending on the oxidizing agents used: HNO_3_ > O_3_ > H_2_O_2_ > O_2_ (air). Mostly carboxyl groups were formed by the oxidative treatments with HNO_3_ and ozone. However, HNO_3_ also causes a decrease of microporosity and of the basic sites on the carbon materials compared to ozone modification, thus O_3_ is the most promising reagent for the formation of oxygen functional groups [108]. Han et al. investigated activated carbons with similar porosity, but different amounts of oxygen groups on the surface. They observed a decrease of the hydrophobicity of the carbon surface due to formation of acidic groups by oxidization with H_2_O_2_. This property change based on the increase of oxygen surface groups made the surface more accessible for the aqueous metal solution during the impregnation process and results in a better platinum dispersion. Nevertheless, less acidic and more thermally stable surface groups avoid the sintering of the metal particles by enhanced metal–carbon interaction [109]. Figueiredo et al. observed different types of oxygen surface groups depending on the oxidizing agent. Oxidative treatment with liquid agents (HNO_3_ or H_2_O_2_) increases the amount of carboxylic groups, while carbonyl and hydroxy surface groups result from modification with O_2_ or N_2_O [110].

**Figure 2:**
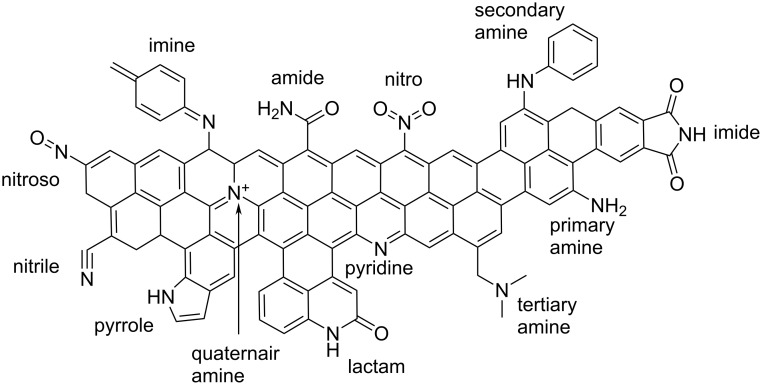
Overview of nitrogen-containing functional groups on the surface of activated carbons. Scheme was drawn according to [10,112].

**Nitrogen-containing surface groups:** Nitrogen-enrichment of activated carbons is possible at the precursor stage or as a modification step after the production of activated carbons and results mostly in a basic character of the prepared materials. Typical nitrogen agents are ammonia, urea or amines. Different types of nitrogen-containing functional groups on activated carbon surfaces are shown in Figure 2 [10,111].

Ammoxidation of activated carbons is a very effective process for the introduction of nitrogen surface groups. The simultaneous oxidation and nitrogenation of the activated carbon samples changes the chemical structure significantly and thus the acid–base character of the materials. Pietrzak et al. observed that the amount of nitrogen in the activated carbons depend on the preparation stage at which the ammoxidation is performed and of the different pre-treated carbon precursors used. The highest nitrogen content in the samples was found by ammoxidation of demineralized coal in the last preparation step after carbonization and activation [10]. Hu and coworkers synthesized nitrogen-doped carbon materials from coconut shell by urea modification and K_2_CO_3_ activation. The carbonized precursor is mixed with urea (1:1 weight ratio), heated and the unreacted urea is removed by a washing step with hot water. Subsequently, the urea-treated samples were activated. The treatment with urea enhanced significantly the nitrogen content in the samples, while the final amount of nitrogen was reduced by the activation step [63,113].

### Activated carbon characterization

Many production methods and possible precursors for activated carbon preparation are known. Also many characterization methods have been reported. In most cases, several characterization methods are used to allow a correlation of the resulting activities and activated carbon properties with the preparation and modification methods. In the following, typical characterization techniques are introduced and the information obtained from the analytical methods is discussed on selected examples.

#### Surface characterization

**Scanning electron microscopy (SEM):** Surface morphology of activated carbons is investigated with scanning electron micrographs. The measurement determines the porosity of a surface area. Cavities or holes can be observed on activated carbon with higher porosity, while smooth surfaces characterize activated carbon with less porosity [71].

Singh et al. showed SEM pictures of activated carbons prepared from the biomass *Arundo donax* with different ratios of KOH as activating reagent. Smooth surfaces were found without KOH, while a high degree of porosity was obtained by the optimum KOH to biomass ratio, resulting in high surface areas [114]. Saka et al. have shown that the external surface of the activated carbons prepared from acorn shell by chemical activation with ZnCl_2_ exhibit cracks and holes in different sizes. They concluded that a porous structure was formed due to the volatization of most of the organic compounds during the carbonization process and the ruptured surface obtained [46]. SEM micrographs also allow the determination of different types of pores. The group of Okman showed SEM investigations on activated carbons prepared from grape seeds by activation with KOH. The sponge-like surface of the activated carbon indicates a microporous structure [56].

**Fourier transmission infrared spectroscopy (FTIR):** Activated carbon consists mainly of carbon atoms, besides different heteroatoms such as oxygen, hydrogen, nitrogen and sulfur. Thus, different functional groups govern the surface of the activated carbons and FTIR provides information on these chemical structures [65]. The spectra are usually recorded between 4000 cm^−1^ and 400 cm^−1^. The most characteristic bands of functional groups on the surface of the activated carbons are ≈3500, 1700, 1610, 1420 and 1140 cm^−1^ which indicate free or intermolecular bonded OH groups, carbonyl (C=O) stretching vibrations of carboxyl groups, ketones or aldehydes, C=C double bonds, aromatic rings and ether C–O stretching bonds, respectively [65,82,115]. Changes of the surface properties due to the modification of the activated carbon samples can also be detected by FTIR measurements.

Shafeeyan et al. investigated new N-containing functional groups on the surface of ammonia treated activated carbon samples such as bands of N–H stretching vibrations (3376–3294 cm^−1^), cyclic amides (1665–1641 cm^−1^), nitriles (2251–2265 cm^−1^) and pyridine-like functionalities (1334–1330 cm^−1^). Simultaneously, a diminished band at about 1700 cm^−1^ was found due to the decomposition of the oxygen-containing surface groups at higher treatment temperatures [115]. The group of Moreno-Castilla investigated the treatment with oxidizing agents (H_2_O_2_ or HNO_3_) or activating reagents by FTIR. They found that the amount of oxygen fixed on the surface of the treated carbons in form of carboxyl groups, ketones, ether groups and carboxyl-carbonate structures is higher using nitric acid compared to hydrogen peroxide [116].

**Nitrogen adsorption–desorption isotherms:** The surface area and pore size distribution of solid catalyst materials can be determined by gas adsorption–desorption measurements at 77 K. To obtain reproducible isotherms from the measurements, a controlled outgassing of the adsorbent with a defined temperature, change in pressure and residual pressure is necessary to remove all physisorbed species from the surface. Different methods can be used for the measurements. The volumetric method with determination of the gas removed from the gas phase and the gravimetric method, where the uptake of the gas by the adsorbent is determined by the increase in mass. In addition, static or dynamic techniques are available for the determination of adsorbed gas [117].

As a result, physisorption isotherms are obtained by plotting the amount of adsorbed gas *n*^a^ in mol g^−1^ against the equilibrium relative pressure (*p*/*p*^0^). The resulting isotherms can be grouped in six different types. Type 1 isotherms are concave to the *p*/*p*^0^ axis and *n*^a^ approaches the limiting value *p*/*p*^0^ → 1. This type is formed by microporous solids with a relatively small external surface, for example, activated carbons or molecular sieve zeolites [117].

Numerous methods are available for calculating surface area, pore size, pore distribution and pore volume by fitting to the isotherms with different assumptions. For example, the Brunauer–Emmet–Teller (BET) method [118] or the Langmuir method [119] for the determination of the surface area of porous materials, the Dubinin–Radushkevich equation [120] for the calculation of microporosity and the Barrett–Joyner–Halenda model [121] for pore size distribution. In addition, the hysteresis between the adsorption and desorption gives information on mesopores. The absence of a hysteresis indicates that there is no or only little mesoporosity [65]. An overview of the pore classification and the concerning pore sizes is shown in Table 2 [117].

**Table 2:** Overview of the pore classification in the context of physisorption [117,122].

Pore type	Pore size

macropores	50 nm
mesopores	2–50 nm
micropores	<2 nm
ultramicropores	<0.7 nm

Sethia et al. investigated the influence of the activation temperature on the porosity and surface area of nitrogen-containing activated carbon samples by N_2_ adsorption measurements. Non-activated carbon samples showed a very low nitrogen uptake indicating small surface areas without pores. A sharp increase of the isotherms at very low relative pressures with subsequent stagnation could be observed with activated carbons prepared between 550 and 650 °C due to a narrowly distributed ultramicroporous structure. The prepared activated carbon sample at 700 °C showed a broader isotherm knee resulting from a wider pore distribution [123]. Kalderis et al. prepared activated carbons from bagasse and rice husk by chemical activation with ZnCl_2_, NaOH and H_3_PO_4_. They observed that the surface area of the activated carbon depends strongly on the activation agent used, the impregnation ratio of the raw material to activation agent, the activation temperature and activation time. ZnCl_2_ showed the best results with surface areas of 674 and 750 m^2^ g^−1^, while the activation with H_3_PO_4_ led to surface areas below 100 m^2^ g^−1^ due to a high retention of phosphates in the carbon structures forming phosphate esters or polymerization byproducts that bind on the solid carbon matrix [30].

**Boehm titrations:** Boehm titrations are used for the determination of acidic or basic surface oxygen functional groups of solid materials. The acidic character is caused by carboxyl groups (R–COOH), lactones (R–OCO), phenolic groups (R–OH) and carbonyl or quinone groups (R=O). Differentiation is possible by titration with different basic solutions NaHCO_3_, Na_2_CO_3_, NaOH, NaOC_2_H_5_, respectively. Titration of the samples with hydrochloric acid determines the basic properties on activated carbon surface in form of pyrone or chromene-like structures and aromatic π electrons [108,124–125].

Nowicki et al. showed different acidic and basic conditions on the surface of the activated carbon materials resulting from different activation methods of cherry stones-based carbons. Activation by carbon dioxide led to basic surface character, while using the chemical activation with KOH results in weakly acidic surface properties of the materials. The temperature used for the activation showed less influence compared to the activation method [52]. Comparison of the amounts of acidic and basic surface groups on treated and untreated activated carbon from bituminous coal investigated Pietrzak. He found that the carbonization process reduced slightly the amount of acidic surface groups, while the amount of basic surface groups remains constant. Nitrogen introduction to the materials led to an obvious decrease of acidic surface groups and simultaneously increase of basic properties [10].

**Phenol, methylene blue and iodine adsorption:** Adsorption capacity and amount of pores of activated carbon materials are determined by using different adsorbates (phenol, methylene blue and iodine). The activated carbons are added to defined methylene blue or phenol solutions and are shaken for a certain time. The concentration of the adsorbates methylene blue and phenol is spectrophotometrically determined at defined absorbance wavelengths. The iodine number is determined by titration with sodium thiosulfate. Generally, the iodine number represents the surface area resulting from the amount of micropores (<1 nm). Mesopores (<1.5 nm) are denoted by methylene blue adsorption, which is also used as model substrate for the adsorption of organic pollutants [51,126]. Phenol adsorption takes place in ultramicropores and micropores with diameters between 0.7 nm and 2 nm and thus, determination of specific surface areas is possible. In addition, phenol is the primarily used liquid-phase reference for adsorption studies. The adsorption capacity of phenol is influenced by oxygen-containing functional groups: basic properties promote the adsorption of phenol by oxidative coupling reactions and acidic functional groups decrease the amount of adsorbed phenol [51,127–128].

Duman and co-workers studied different pyrolysis temperatures and activation times with ZnCl_2_ for the preparation of highly porous activated carbon from fruit stones and nutshells. They found that both conditions influence strongly the adsorption of phenol and methylene blue on the nutshell-based carbons. It is essential to find the optimum activation time, since shorter treatments (6 h) do not lead to a porous structure and longer activation times (24 h) causes a collapsing of the structure [51]. Song et al. used methylene blue adsorption and iodine number to investigate the surface area and porosity of the AC prepared from corncob by physical (steam) and chemical (KOH) activation with different carbonization and activation conditions. Chemical activation showed obviously higher surface areas compared to steam activation [71].

**Temperature-programmed desorption (TPD):** TPD is used for the study of surface oxides due to the thermal stability of the surface groups. The samples were heated in an inert gas atmosphere or in vacuum with a constant heating rate and the evolved gases were determined by mass spectrometry. In general, each type of surface group decomposes to a defined product such as CO_2_ from carboxylic acid or lactones and CO from carbonyl, hydroxide, phenol, ether or quinone groups and thus, information on the amounts of oxygen-containing surface groups are obtained. Nevertheless, the decomposition products are not always clearly assignable, since two adjacent carboxyl groups form primarily the anhydride followed by decomposition to CO and CO_2_ [15,110,124].

Capart and co-workers investigated the activated carbons produced from wood and the basic wood components lignin, cellulose and xylan by KOH activation with TPD. The different prepared materials showed no significant difference of the surface functionalities. All TPD spectra show a water peak at about 500 K according to dehydration of carboxylic acid during formation of anhydrides, a CO peak at 900 K due to decomposition of carboxylic anhydrides and a CO_2_ peak at about 500 K resulting from carboxylic acids [15]. Figueiredo et al. examined the influence of oxidative treatments on activated carbon by TPD studies. Enhanced CO and CO_2_ peaks found by TPD indicate an increasing amount of oxygen-containing surface groups on the activated carbon materials. They could observe that gas phase oxidation led to a higher amount of mainly hydroxy and carbonyl groups, whereas liquid phase treatment with nitric acid results in an increase of carboxylic acid groups [110]. Lillo-Ródenas et al. used TPD for the determination of the released gases during the activation process of anthracite with sodium or potassium hydroxide and thus for clarification of the activation mechanism with hydroxides. The reaction of carbon precursor and metal hydroxide at lower temperatures (Na: 570 °C and K: 400 °C) lead to the formation of hydrogen and metal carbonates as well as metallic metal or M_2_O (M = Na or K). The absence of carbon dioxide at these temperatures suggests that the carbonates are not formed by reaction of hydroxides with CO_2_. At higher temperatures, CO and CO_2_ were found due to the decomposition of the metal carbonates. Moreover, they found that no porosity of the resulting materials was formed by using a metal carbonate instead of hydroxide as activation reagent [8,14]. Pis and co-workers used the TPD method for evaluation of the thermal stability of introduced nitrogen functionalities by modification with ammonia due to the evolution of NH_3_ and HCN. Depending on the modification temperature, nitrogen is incorporated mainly to aromatic rings at higher temperatures, while less temperature-stable amide-like functionalities were formed at lower temperatures [129].

### Composition of activated carbon

**Elemental composition by CHNS/O:** The elemental composition is determined for starting materials as well as for the resulting activated carbon materials. Thereby, the influence of the different preparation, activation and modification methods on the carbon or heteroatom content can be compared.

Nowicki et al. found that physical activation of cherry stones results in higher carbon contents and simultaneously lower hydrogen and oxygen amounts, while chemical activation show only smaller changes of the elemental composition with exception of a significant decrease of the nitrogen content [52]. Pietrzak examined the different elemental compositions of the bituminous coal based materials after carbonization, activation and ammoxidation for the enrichment of nitrogen. He found that the carbonization step causes an increase of the carbon content, a decrease of oxygen and hydrogen and the nitrogen amount stays almost constant. The activation process leads to an increase of oxygen, while all other elements decrease. Thus, the activation reagent (KOH) oxidizes the initial material during this step. As expected, the ammoxidation results in an enrichment of nitrogen [10].

**Scanning electron microscopy with energy-dispersive X-ray (SEM-EDX):** An energy-dispersive X-ray detector allows the investigation of the composition of the materials and of the distribution of elements on the investigated materials by EDX mapping.

Ternero-Hidalgo et al. found by SEM-EDX investigations that the treatment of olive stones-based carbons with H_3_PO_4_ during chemical activation or modification with HNO_3_ occurs uniformly on the entire surface, since the heteroatoms (nitrogen and phosphorous) are quite homogeneously distributed on the surface of the activated carbons [130].

**X-ray photoelectron spectroscopy (XPS or ESCA):** X-ray photoelectron spectroscopy determines the chemical state of elements and the composition of the sample on the upper surface layer (only few atomic layers). X-ray irradiation (Mg Kα or Al Kα) excite core electrons to leave the atoms and their kinetic energies are measured. The characteristic binding energies are calculated from the measured kinetic energies. After baseline subtraction, the curves are fitted to Gaussian and Lorenztian peak shapes with different proportions. For calibration of the XPS method, the carbon 1s electron binding energy was referenced at 284.6 eV [10,124,131–132].

Pietrzak investigated with XPS different methods for the enrichment of bituminous coal by ammoxidation. The greatest enrichment of nitrogen occurs by using the ammoxidation as last step after carbonization and activation of the bituminous coal. The measurements showed that nitrogen is introduced in the activated carbon as amines, imines, amides, pyridine nitrogen and pyrrole nitrogen or as oxidized nitrogen species, e.g., pyridine-*N*-oxides [10]. Díaz-Terán et al. examined the surface of the samples (surface groups, chemical state of the elements, metal content and distribution) during the activation process of lignocellulosic precursor with KOH by XPS. They observed that the oxygen on the surface of the material is associated with potassium as carbonates or oxides [131]. Cordero and co-workers observed by XPS that different amounts and species of nitrogen-containing surface groups are obtained by HNO_3_ treatment of olive stones-based activated carbon depending on the activation method. Chemical activation by H_3_PO_4_ form a higher amount of N-containing surface groups, mainly as nitro groups, compared to physical activation with CO_2_, whereby only less oxidized nitrogen species were formed. They concluded that the phosphorous species in the carbons could be responsible for the examined difference [130].

**X-ray diffraction (XRD):** X-ray diffraction gives information on the crystallinity or amorphicity of activated carbons. Comparison of resulting XRD patterns with the crystallographic databases clarify that partially graphitic structures are available in the activated carbon materials. In addition, XRD can be used for investigations of the activation process by detecting crystalline intermediates of the activation reagents.

Liang and co-workers found in the activated carbon prepared by microwave-induced ZnCl_2_ activation of wood broad peaks at about 23° and 44° due to the (002) and (100) reflexes of graphite. Thus, they concluded the formation of a carbon structure with randomly orientated graphitic carbon layers. Calculation of *d* values by the Bragg equation allows the comparison of the layer distances found in the activated carbon samples with the values of graphite (0.335 nm). The prepared wood-based activated carbon show higher *d* values between 0.365 nm and 0.375 nm [104]. Singh et al. received very similar results by KOH activation of biomass *Arundo donax* with broad peaks at 23° and 43°. Moreover, they found that the intensities of the reflections are enhanced by an increasing KOH to biomass ratio, indicating that KOH promotes the graphitization of the prepared activated carbons [114]. Díaz-Terán et al. used XRD to detect the development of crystalline compounds during the activation of a lignocellulosic precursor with KOH. They found the formation of K_2_CO_3_ during this step, which increases with the pyrolysis temperature and is responsible for the pore development [131].

**Thermogravimetric analysis (TGA):** TGA provides information on the weight loss of the starting materials during the heating process due to, e.g., decomposition.

Kumar et al. found by using TGA that the activation process of the used nutshells by ZnCl_2_ proceeds in three steps. In the beginning, the organic matter decomposes, followed by a further decomposition of the obtained intermediates and the activating reagent. At least, the char reacts with ZnCl_2_ and the pores open [133]. Kalderis and co-workers observed also three steps in the study of the thermal behavior of rise husk and bagasse. The first step (≈100 °C) shows a weight loss due to the moisture of the samples. Further heating to about 370 °C led to decomposition of the main components cellulose, hemicellulose and lignin and the loss of the volatile matter is responsible for the decrease in weight. Higher temperatures result in carbonization of the residues and gaseous products or tars are formed associated with another weight loss [30].

**Ash content:** The total ash content is determined by using ASTM standards. A defined amount of sample is weighed in a porcelain basin, heated in a muffle furnace to a given final temperature and for a certain time. After cooling to room temperature in a desiccator, the sample is reweighed. The ash content is calculated using the following equation [30,50]:

[4] $$\text{ash content }\left[\text{\%}\right]\text{ = }\frac{\text{remaining solids weight }\left[\text{g}\right]}{\text{original materials weight }\left[\text{g}\right]}\;\cdot \text{ 100}$$

### Metal loading methods

The macroscopic distribution of a metal on the support, the size of the metal crystallites on the support surface and the oxidation state of the metal species determine the catalytic performance of a supported metal catalyst [1].

#### Impregnation und adsorption

The impregnation method is comparatively simple and thus widely used in the preparation of supported catalysts. Three different methods are available: wet impregnation, incipient-wetness impregnation and ionic adsorption. Using these methods for the preparation of supported metal catalysts, the interaction between the precursor and supporting material and its pore systems shows the greatest influence on the dispersion of the metal on the support and thereby limits the metal loading [134]. Various factors play also a role in the distribution of the metal precursor on the support: type of metal compound, solvent used and pH of the solution [135–136].

For the wet impregnation, the supporting material is added to a large excess of solution with the metal precursor. Thereby, larger catalyst particles with an egg-shell distribution of the metal are formed due to the deposition of the metal precursor on the outside of the support without using the pore system. To use the pore system of the supporting materials and obtain smaller catalyst particles with a uniform metal loading, the incipient-wetness impregnation method was developed. The metal precursor is dissolved in exactly that amount of solvent, which is necessary to fill the pores of the supporting material, whereby a closer contact of the metal precursor and the support is guaranteed [134]. After the impregnation, the solvent is removed by a drying step and reduction of the metal precursor is necessary to form the active metal on carbon catalysts by adding a reducing agent, such as hydrogen, formaldehyde or sodium borohydride [1]. Is the drying step replaced by filtration of the large excess of solution, the method is called ionic adsorption [134,137].

#### Deposition

The deposition of a precursor of an active component from an excess of solution onto a support by a chemical reaction is called deposition precipitation. It is necessary to have enough carboxylic acid groups available on the support to obtain a high dispersion of the metal.

The increase of pH, the change of valency of the metal ion (electroless plating) or the removal of a stabilizing ligand of the metal ion allow the deposition precipitation from a suspension. Compared to the impregnation/adsorption method, several advantages can be obtained by this method: reproducibility, high metal loadings, high metal dispersion at high metal loadings and uniform distribution of the metal over the support [1,134,137].

### Summary – Influence of the preparation parameters on the material properties

Each step in the preparation of a carbon-supported catalyst influences its final properties such as the surface area, the pore size distribution, the attrition resistance, the ash content and the surface chemistry of the carbon materials and thus the performance of the catalyst [1].

The choice of starting material influence the particle size distribution, morphology and the attrition resistance of the carbon material. These properties determine the filterability and activity of the prepared supported metal catalysts. A smaller particle size leads to an increased geometrical area and thus to a higher catalytic activity of the catalyst, while larger particles enhance the filterability of the materials during catalyst recycling [1].

The pore size distribution of the carbon-based supports, influenced by the precursor material and the preparation and activation techniques used, determines the available surface area for the impregnation with catalytically active metal particles. In addition, the porosity influences the access of reactants to the active supported metal particles, thereby affecting the catalytic activity [1,138].

The surface chemistry, in particular oxygen-containing surface groups of the activated carbon support, influence the preparation of the catalyst and the resulting activity. The oxygen functionalities determine the acid–base character for adsorption of ionic species or the redox properties of the materials for deposition of the metal particles by redox reaction with carbon. Acidic oxygen functional groups (carboxyl or lactone groups) reduce the hydrophobicity of the carbon and thereby increase the surface accessibility to the aqueous metal precursor solution. Moreover, they ensure highly dispersed metal crystallites and stabilize them [1,139–141].

Nevertheless, a rational design of carbon-supported catalyst materials is still difficult. Empiricism, experience and precise procedures in practical production are important elements for the reproducible preparation of active and stable heterogeneous supported catalysts.

## Conclusion

A brief overview of the preparation of metal on carbon catalysts is given. The activated carbon materials preparation is summarized by choice of precursor materials, available production and modification methods and possible characterization techniques. Finally, different metal loading methods are shown.

In summary, each step in the preparation of a carbon-supported catalyst influences its final properties such as the surface area, the pore size distribution, the attrition resistance, the ash content and the surface chemistry of the carbon materials and thus the amount, dispersion and size of the loaded metal. Accordingly, the catalyst must be developed individually for the different applications according to the desired requirements based on empirical and theoretical knowledge.
